# Harmonizing Dentofacial Balance: A Comprehensive Exploration of Class 1 Malocclusion Resolution With Impacted Maxillary Central Incisor Management

**DOI:** 10.7759/cureus.51582

**Published:** 2024-01-03

**Authors:** Nishu Agarwal, Pallavi Daigavane, Ranjit Kamble, Dhwani Suchak, Mrudula Shinde

**Affiliations:** 1 Orthodontics and Dentofacial Orthopaedics, Sharad Pawar Dental College and Hospital, Datta Meghe Institute of Higher Education and Research, Wardha, IND

**Keywords:** class 1 malocclusion, supernumerary tooth, comprehensive treatment, impacted teeth, multidisciplinary approach

## Abstract

Permanent maxillary central incisor impaction is generally a rare phenomenon. Because the anterior teeth have a huge impact on a patient's facial aesthetics, missing anterior teeth are of major concern to patients who seek orthodontic treatment. However, correcting this type of malocclusion poses a challenge to the orthodontist. This case report of a 16-year-old male patient with an impacted maxillary right central incisor takes us through a series of events that are necessary to treat this type of malocclusion. The presenting case also had an impacted supernumerary tooth which was the primary cause for permanent tooth impaction. A combined treatment approach is usually needed to manage this type of case involving both the orthodontist and the surgeon.

## Introduction

The main aim of orthodontic treatment focuses on achieving a healthy periodontium and a balanced functional occlusion which results in improved facial aesthetics and overall appearance of the patient. Surgical intervention is needed in cases where a permanent tooth fails to erupt in the oral cavity. Various factors associated with this impaction are thick mucosa covering the tooth, association of a cyst, supernumerary tooth associated with the impacted permanent tooth, and no space availability due to crowding in the dental arch [1].

Eruption of a tooth into the oral cavity occurs when two-thirds of the root formation is completed. If a tooth does not erupt in the age range where it should erupt, it is considered to be impacted. Surgical intervention and orthodontic treatment are usually necessary to bring the teeth into their normal alignment in the dental arch [2].

Prevalence of impaction of the maxillary central incisor is found to be 0.03-0.20%. Though this is a rare occurrence, it has a huge impact on a patient's facial aesthetics, speech, and psychology [3]. When the tooth is covered by a thick mucosa that is hindering its eruption, surgical exposure only will allow the tooth to erupt on its own, provided there is space in the dental arch. However, when other conditions as mentioned above are involved, a combination of surgical intervention and orthodontic therapy is needed to achieve the desired result [4]. If favorable conditions are not present for the eruption of the impacted tooth, other treatment options include prosthetic rehabilitation depending on the patient's case. In these cases, interdisciplinary management is needed [5].

## Case presentation

**Figure 1:**
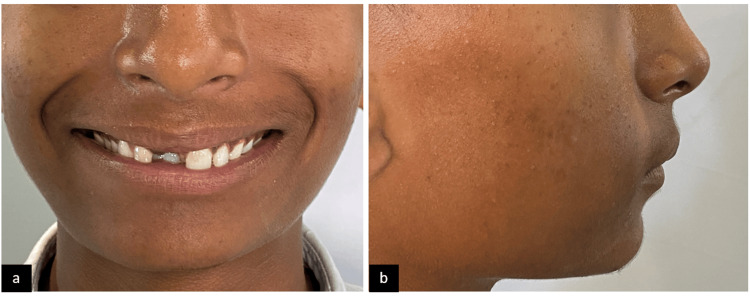
Pre-treatment extraoral photographs: (a) frontal smiling and (b) profile

**Figure 2:**
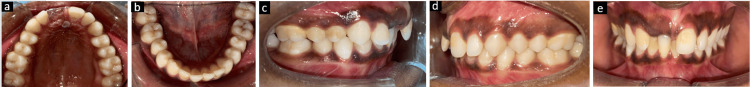
Pre-treatment intraoral photographs: (a) maxillary arch, (b) mandibular arch, (c) right molars in occlusion, (d) left molars in occlusion, and (e) anteriors in occlusion

**Figure 3:**
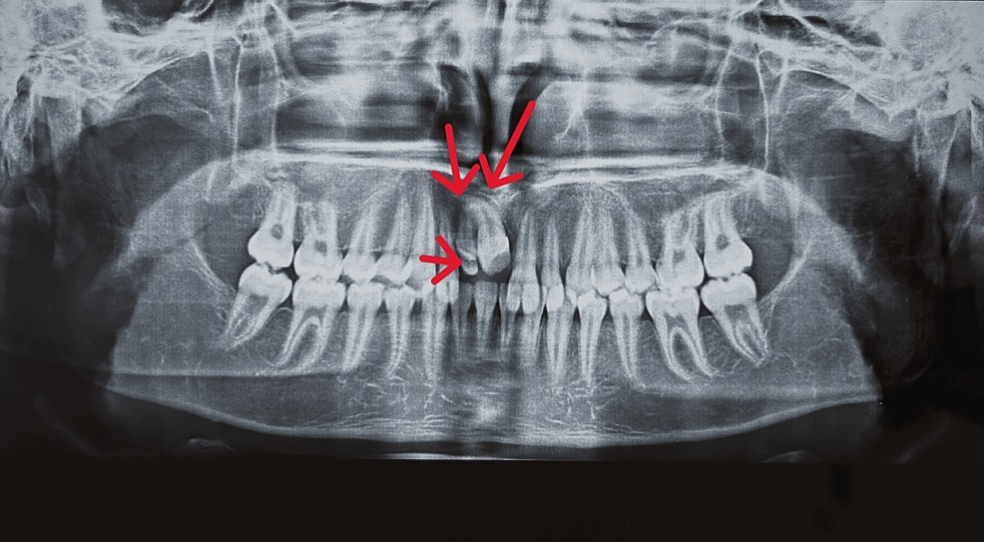
Pre-treatment panoramic radiograph

The presented case is of a 16-year-old male patient with a main complaint of missing upper front teeth. No significant medical history was present. The patient had an orthognathic facial profile (Figure 1). Upon clinical examination, molars were in class 1 relationship with missing 11 (Figure 2). A panoramic radiograph was taken to assess the position and prognosis of the impacted maxillary central incisor (Figure 3).

**Figure 4:**
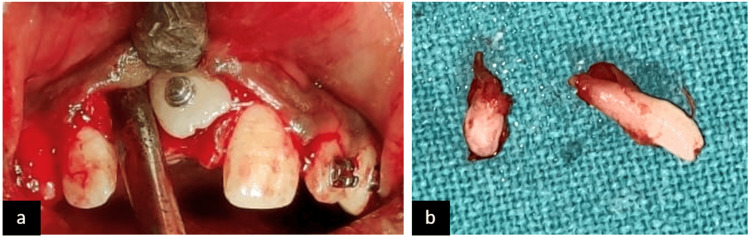
Surgical procedure: (a) surgical exposure of the impacted tooth and (b) extracted two supernumerary teeth

After assessment of the case, an association of two supernumerary teeth was found with 11, and formulation of a treatment plan was done which included extraction of the supernumerary tooth and surgical exposure of the impacted tooth (Figure 4). The amount of space available in the dental arch was similar to that of the impacted tooth, i.e., 8 mm.

**Figure 5:**
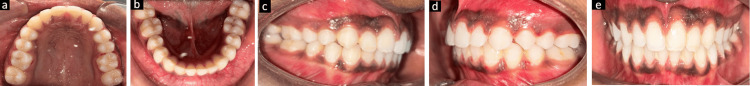
Post-treatment intraoral photographs: (a) maxillary arch, (b) mandibular arch, (c) right molars in occlusion, (d) left molars in occlusion, and (e) anteriors in occlusion

**Figure 6:**
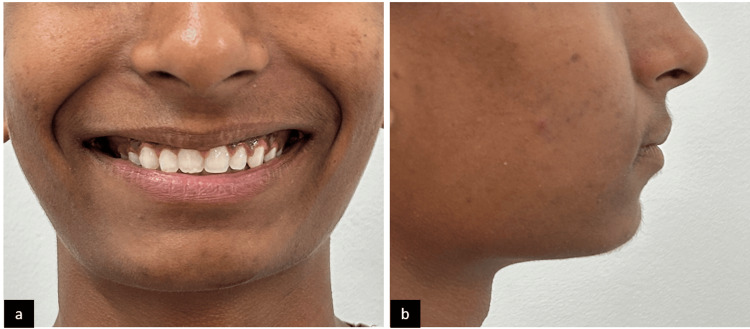
Post-treatment extraoral photographs: (a) frontal smiling and (b) profile

**Figure 7:**
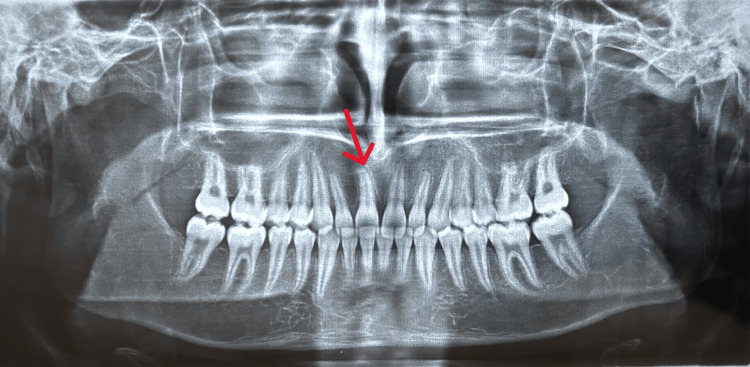
Post-treatment panoramic radiograph

Bonding of both the maxillary and the mandibular arch was carried out. Initial leveling and alignment were done using nickel-titanium (NiTi) wires. Surgical exposure was carried out with 11, and extraction of the supernumerary tooth was done. A traction force was applied to the impacted tooth with a lingual button which was bonded onto the labial surface for forced eruption into the dental arch. After the tooth was visible in the oral cavity, a piggyback wiring was given along with a 17x25" NiTi wire to align the tooth. The treatment took about 13 months to complete, and the patient showed satisfaction with the result (Figure 5 and Figure 6). Post-treatment panoramic radiograph showed adequate bone support to the tooth (Figure 7).

## Discussion

Unerupted teeth in the anterior region can have a psychological impact on the patients. The type of treatment for these types of cases is important. Extraction of a tooth may lead to loss of the surrounding alveolar bone, so if preservation of the tooth is possible, the clinician should attempt it. The goal of an orthodontist is to preserve and align the impacted tooth, if possible, to maintain occlusal harmony and improve patients' facial aesthetics, reducing its adverse effects on patients' psychology [1].

Several factors are to be considered if there is the presence of an impacted tooth: dilaceration of the root if realignment is possible in the available space and direction, transposition, and angulation of the tooth. The extent of dilaceration, the formation of the root, and the tooth position all influence the rate of success of the impacted dilacerated tooth. If a dilacerated tooth is placed at a lower position with an obtuse angle and root formation is not complete, it will have a better prognosis for orthodontic traction [6]. The cause of dilaceration that usually leads to impaction is due to trauma during the deciduous dentition period [7].

In cases of tooth transposition, it might be difficult to treat because of the less width of the buccolingual bone. These types of cases are usually associated with trauma [8]. Many patients opt for a prosthetic replacement for the impacted tooth rather than undergoing surgical exposure. In these cases, the combined treatment plan should be formulated by both the orthodontist and the prosthodontist to improve patients' facial appearance [9,10]. In a study conducted by Khera et al., a closed eruption technique for surgical exposure was used [11]. Becker in his study concluded that the closed eruption of impacted teeth gave better results [12]. Nisa et al. [13] in their study after exposure to the impacted tooth used traction force for the eruption similar to this case. In the presented case report, the availability of space was enough to bring the impacted tooth into normal alignment; hence, no extractions were carried out and alignment of the tooth was achieved.

## Conclusions

Impacted central incisor is a rare occurrence, but addressing it is of immense importance. Treatment method and treatment time depend upon the location of the tooth and various other factors discussed above. Early intervention is necessary to treat this type of malocclusion.
